# (*E*)-3-(4-Hydr­oxy-3-methoxy­benzyl­idene)-4-(4-hydroxy­phen­yl)pyrrolidin-2-one

**DOI:** 10.1107/S1600536808008581

**Published:** 2008-04-10

**Authors:** Yi-Feng Zhou, Xiao-Bing Wang, Jin Qi, Bo-Yang Yu

**Affiliations:** a Department of Complex Prescription of TCM, China Pharmaceutical University, Nanjing 210038, People’s Republic of China; b Department of Natural Medicinal Chemistry, China Pharmaceutical University, Nanjing, 210009, People’s Republic of China

## Abstract

The title compound, C_18_H_17_NO_4_, was isolated from an ethanol extract of *Ophiopogon japonicus*. The dihedral angle between the 4-hydroxy-3-methoxyphenyl ring and the pyrrolidine ring is 17.4 (1)°. The 4-hydroxyphenyl ring makes a dihedral angle of 69.74 (6)° with the least-squares plane through the 4-hydroxy-3-methoxyphenyl ring and the pyrrolidine ring. The conformation of the pyrrolidine fragment is similar to a T-form. The crystal structure is stabilized by inter­molecular N—H⋯O and O—H⋯O hydrogen bonds.

## Related literature

For the chemical components and pharmacological properties of the plant *Ophiopogon japonicus*, see: Anh *et al.* (2003 ▶); Kou *et al.* (2005 ▶) & Yu (2007 ▶). For related literature, see: Bernstein *et al.* (1995 ▶).
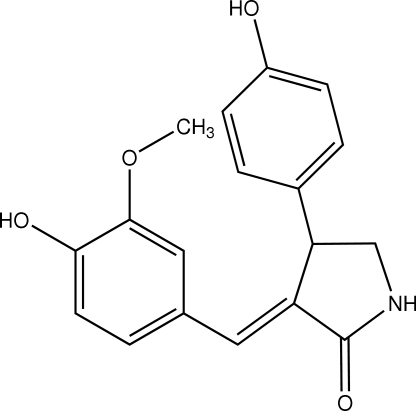


## Experimental

### 

#### Crystal data

C_18_H_17_NO_4_
*M* *_r_* = 311.33
Monoclinic, 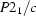

*a* = 6.388 (1) Å
*b* = 14.520 (2) Å
*c* = 16.880 (2) Å
β = 96.514 (2)°
*V* = 1555.6 (4) Å^3^
*Z* = 4
Mo *K*α radiation
μ = 0.09 mm^−1^
*T* = 298 (2) K
0.47 × 0.42 × 0.35 mm

#### Data collection

Bruker SMART CCD area-detector diffractometer
Absorption correction: multi-scan (**SADABS**; Sheldrick, 1999 ▶) *T*_min_ = 0.954, *T*_max_ = 0.969
9225 measured reflections
3387 independent reflections
1756 reflections with *I* > 2σ(*I*)
*R*_int_ = 0.041

#### Refinement

*R*[*F* ^2^ > 2σ(*F* ^2^)] = 0.047
*wR*(*F* ^2^) = 0.137
*S* = 1.02
3387 reflections
209 parameters
H-atom parameters constrained
Δρ_max_ = 0.22 e Å^−3^
Δρ_min_ = −0.20 e Å^−3^

### 

Data collection: *SMART* (Bruker, 2001 ▶); cell refinement: *SAINT*; data reduction: *SAINT* (Bruker, 2001 ▶); program(s) used to solve structure: *SHELXS97* (Sheldrick, 2008 ▶); program(s) used to refine structure: *SHELXL97* (Sheldrick, 2008 ▶); molecular graphics: *SHELXTL* (Sheldrick, 2008 ▶); software used to prepare material for publication: *SHELXTL*.

## Supplementary Material

Crystal structure: contains datablocks I, global. DOI: 10.1107/S1600536808008581/lx2053sup1.cif

Structure factors: contains datablocks I. DOI: 10.1107/S1600536808008581/lx2053Isup2.hkl

Additional supplementary materials:  crystallographic information; 3D view; checkCIF report

## Figures and Tables

**Table 1:** Hydrogen-bond geometry (Å, °)

*D*—H⋯*A*	*D*—H	H⋯*A*	*D*⋯*A*	*D*—H⋯*A*
N—H1⋯O1^i^	0.86	2.09	2.948 (2)	172
O2—H2⋯O1^ii^	0.82	1.95	2.675 (2)	147
O4—H4⋯O2^iii^	0.82	2.00	2.721 (2)	147

Symmetry codes: (i) 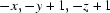; (ii) 
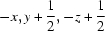
; (iii) 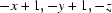.

**Fig. 1.**
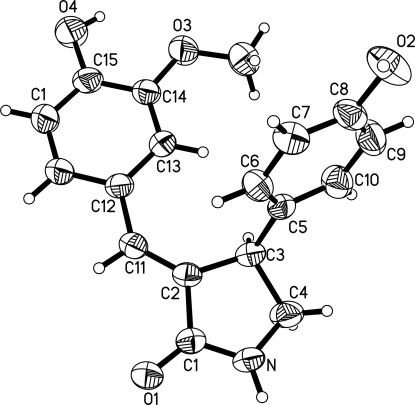
A drawing of the title molecule with the atom-numbering scheme. Displacement ellipsoids are drawn at the 50% probability level.

**Fig. 2.**
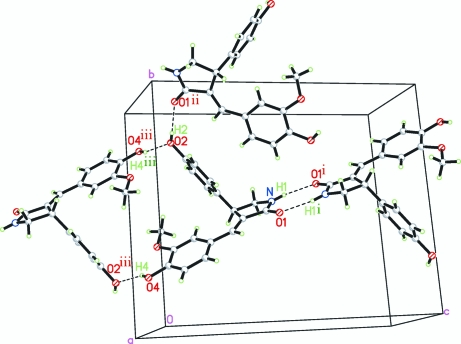
N—H···O and O—H···O hydrogen bond interactions (dotted lines) in the title compound. [Symmetry code: (i) -x, -y+1, -z+1; (ii) -x, y+1/2, -z+1/2; (iii) -x+1, -y+1, -z.]

## supplementary crystallographic information

### Comment

The plant of *Ophiopogon japonicus* (*L*. f.) Ker-Gawl.(Liliaceae) is widely distributed in South-east Asia, especially in most areaof China, and its tuber root as a famous traditional medicine are widely usedin China to cure acute and chronic inflammation and cardiovascular diseasesincluding thrombotic diseases for thousands of years (Yu, 2007; Kou, *et al.*, 2005). Chemical studies have shown that this plant includes steroidal saponins, homoisoflavonoids andmonoterpene glycosides *etc* (Anh, *et al.*, 2003). Herein we report the molecular and crystal structure of the title compound (Fig.1), which was isolated from an ethanol extract of the plant of *Ophiopogon japonicus*.

The main components of the title compound were two aromatic rings, A(C5—C10) and B(C12—C17) and a pyrrolidine ring C(N1/C1—C4) as shown in Fig. 1. Fig. 2 presents the packing diagram of the title compound. Paired molecules at the inversional position assembled *via* supromolecular sython *R*_2_^2^(8) (Bernstein, *et al.*, 1995) which consist of hydrogen bonds N1—H1···O1^i^, O2—H2···O1^ii^ and O4—H4···O2^iii^ (Symmetry code as in Fig. 2.).

### Experimental

Material from the dried subterranean parts of *Ophiopogon japonicus* (*L*. f.) Ker-Gawl.(Liliaceae) (40 kg),collected from Sichuan Province in China, was extracted with hot 60% EtOH (3×3 h) under refluxing. The concentrated extract was subjected to D-101 macroporous resin column chromatography eluted successively with EtOH-H_2_O(0:100, 30:70, 90:100) to give three fractions (I-III). The concentratedresidue of fraction III (EtOH-H_2_O, 90:10) (330 g) was further dissolved in water, and extractedwith EtOAc and n-BuOH successively. The EtOAc extract (107 g) was loaded onto a silica-gel column (200–300 mesh, 600 g) eluted with a gradientof 100% CHCl_3_ to CHCl_3_—MeOH (50:50) to give 18 fractions,which was pooled by common thin-layer chromatography characteristics. Fraction9 was subjected to repeated chromatography over silica-gel and Sephadex LH-20columns, gave compound (I) (yield 6 mg, m.p. 518 K). Prismatic crystalssuitable for X-ray studies were grown from MeOH by slow evaporation at roomtemperature.

### Refinement

(type here to add refinement details)

### Crystal data

**Table:**

C_18_H_17_NO_4_	*F*_000_ = 656
*M**_r_* = 311.33	*D*_x_ = 1.329 Mg m^−^^3^
Monoclinic, *P*2_1_/*c*	Mo *K*α radiation λ = 0.71073 Å
Hall symbol: -P 2ybc	Cell parameters from 1858 reflections
*a* = 6.388 (1) Å	θ = 2.4–23.1º
*b* = 14.520 (2) Å	µ = 0.09 mm^−^^1^
*c* = 16.880 (2) Å	*T* = 298 (2) K
β = 96.514 (2)º	Block, colourless
*V* = 1555.6 (4) Å^3^	0.47 × 0.42 × 0.35 mm
*Z* = 4	

### Data collection

**Table:**

Bruker SMART CCD area-detector diffractometer	3387 independent reflections
Radiation source: fine-focus sealed tube	1756 reflections with *I* > 2σ(*I*)
Monochromator: graphite	*R*_int_ = 0.041
Detector resolution: 10.0 pixels mm^-1^	θ_max_ = 27.0º
*T* = 298(2) K	θ_min_ = 1.9º
φ and ω scans	*h* = −8→7
Absorption correction: multi-scan(*SADABS*; Sheldrick, 1999)	*k* = −13→18
*T*_min_ = 0.954, *T*_max_ = 0.969	*l* = −20→21
9225 measured reflections	

### Refinement

**Table:**

Refinement on *F*^2^	Secondary atom site location: difference Fourier map
Least-squares matrix: full	Hydrogen site location: inferred from neighbouring sites
*R*[*F*^2^ > 2σ(*F*^2^)] = 0.047	H-atom parameters constrained
*wR*(*F*^2^) = 0.137	*w* = 1/[σ^2^(*F*_o_^2^) + (0.0524*P*)^2^ + 0.295*P*] where *P* = (*F*_o_^2^ + 2*F*_c_^2^)/3
*S* = 1.02	(Δ/σ)_max_ < 0.000
3387 reflections	Δρ_max_ = 0.22 e Å^−^^3^
209 parameters	Δρ_min_ = −0.20 e Å^−^^3^
Primary atom site location: structure-invariant direct methods	Extinction correction: none

### Special details

**Table:**

Geometry. All esds (except the esd in the dihedral angle between two l.s. planes) are estimated using the full covariance matrix. The cell esds are taken into account individually in the estimation of esds in distances, angles and torsion angles; correlations between esds in cell parameters are only used when they are defined by crystal symmetry. An approximate (isotropic) treatment of cell esds is used for estimating esds involving l.s. planes.
Refinement. Refinement of F^2^ against ALL reflections. The weighted R-factor wR and goodness of fit S are based on F^2^, conventional R-factors R are based on F, with F set to zero for negative F^2^. The threshold expression of F^2^ > 2sigma(F^2^) is used only for calculating R-factors(gt) etc. and is not relevant to the choice of reflections for refinement. R-factors based on F^2^ are statistically about twice as large as those based on F, and R- factors based on ALL data will be even larger.

### Fractional atomic coordinates and isotropic or equivalent isotropic displacement parameters (Å^2^)

**Table:**

	*x*	*y*	*z*	*U*_iso_*/*U*_eq_	
N	0.1955 (3)	0.50621 (13)	0.43227 (10)	0.0407 (5)	
H1	0.1694	0.5304	0.4766	0.049*	
O1	−0.1146 (2)	0.42888 (11)	0.40852 (9)	0.0482 (4)	
O2	0.3756 (3)	0.78432 (11)	0.09921 (11)	0.0678 (6)	
H2	0.2621	0.8112	0.0921	0.102*	
O3	0.5094 (3)	0.35253 (12)	0.04340 (10)	0.0600 (5)	
O4	0.2796 (3)	0.20997 (13)	−0.01836 (11)	0.0762 (6)	
H4	0.3957	0.2277	−0.0283	0.114*	
C1	0.0580 (4)	0.45390 (14)	0.38830 (13)	0.0372 (5)	
C2	0.1463 (3)	0.43166 (15)	0.31329 (12)	0.0358 (5)	
C3	0.3493 (3)	0.48408 (15)	0.31322 (12)	0.0382 (5)	
H3	0.4610	0.4408	0.3031	0.046*	
C4	0.3928 (4)	0.5193 (2)	0.39970 (13)	0.0536 (7)	
H4A	0.5045	0.4840	0.4294	0.064*	
H4B	0.4325	0.5838	0.4008	0.064*	
C5	0.3457 (3)	0.56253 (15)	0.25340 (12)	0.0365 (5)	
C6	0.1668 (4)	0.59272 (15)	0.20783 (14)	0.0433 (6)	
H6	0.0395	0.5631	0.2121	0.052*	
C7	0.1720 (4)	0.66655 (16)	0.15552 (14)	0.0472 (6)	
H7	0.0495	0.6858	0.1250	0.057*	
C8	0.3594 (4)	0.71071 (16)	0.14939 (14)	0.0476 (6)	
C9	0.5397 (4)	0.68061 (18)	0.19335 (17)	0.0562 (7)	
H9	0.6671	0.7098	0.1884	0.067*	
C10	0.5334 (4)	0.60767 (17)	0.24462 (15)	0.0523 (7)	
H10	0.6572	0.5880	0.2741	0.063*	
C11	0.0515 (4)	0.37125 (15)	0.26114 (13)	0.0401 (6)	
H11	−0.0771	0.3493	0.2741	0.048*	
C12	0.1159 (4)	0.33400 (15)	0.18741 (13)	0.0396 (6)	
C13	0.2897 (4)	0.36572 (15)	0.15170 (13)	0.0419 (6)	
H13	0.3690	0.4145	0.1747	0.050*	
C14	0.3449 (4)	0.32596 (16)	0.08338 (13)	0.0431 (6)	
C15	0.2293 (4)	0.25225 (17)	0.04909 (14)	0.0507 (7)	
C16	0.0567 (4)	0.22166 (18)	0.08261 (15)	0.0619 (8)	
H16	−0.0229	0.1732	0.0591	0.074*	
C17	−0.0002 (4)	0.26206 (17)	0.15097 (14)	0.0536 (7)	
H17	−0.1182	0.2407	0.1729	0.064*	
C18	0.6343 (4)	0.4276 (2)	0.07462 (17)	0.0672 (8)	
H18A	0.5482	0.4818	0.0749	0.101*	
H18B	0.7457	0.4382	0.0420	0.101*	
H18C	0.6937	0.4137	0.1281	0.101*	

### Atomic displacement parameters (Å^2^)

**Table:**

	*U*^11^	*U*^22^	*U*^33^	*U*^12^	*U*^13^	*U*^23^
N	0.0424 (11)	0.0505 (12)	0.0299 (10)	0.0051 (9)	0.0069 (8)	−0.0053 (9)
O1	0.0476 (10)	0.0547 (11)	0.0451 (10)	−0.0041 (8)	0.0177 (8)	−0.0079 (8)
O2	0.0807 (14)	0.0491 (11)	0.0812 (14)	0.0105 (10)	0.0418 (11)	0.0189 (10)
O3	0.0623 (11)	0.0676 (12)	0.0549 (11)	−0.0166 (10)	0.0276 (9)	−0.0155 (9)
O4	0.0992 (15)	0.0755 (13)	0.0612 (12)	−0.0284 (11)	0.0411 (11)	−0.0326 (10)
C1	0.0417 (13)	0.0371 (13)	0.0333 (12)	0.0044 (11)	0.0064 (10)	−0.0001 (10)
C2	0.0380 (12)	0.0397 (13)	0.0300 (12)	0.0038 (10)	0.0053 (10)	0.0047 (10)
C3	0.0396 (13)	0.0456 (14)	0.0298 (12)	0.0048 (11)	0.0053 (10)	−0.0016 (10)
C4	0.0417 (14)	0.085 (2)	0.0340 (14)	−0.0061 (13)	0.0032 (11)	−0.0047 (13)
C5	0.0387 (13)	0.0396 (13)	0.0319 (12)	−0.0006 (10)	0.0071 (10)	−0.0053 (10)
C6	0.0380 (13)	0.0444 (14)	0.0483 (15)	−0.0005 (11)	0.0087 (11)	0.0033 (11)
C7	0.0463 (15)	0.0490 (15)	0.0466 (15)	0.0082 (12)	0.0070 (12)	0.0062 (12)
C8	0.0598 (17)	0.0367 (14)	0.0506 (15)	0.0017 (12)	0.0251 (13)	0.0006 (11)
C9	0.0492 (16)	0.0550 (17)	0.0660 (18)	−0.0134 (13)	0.0137 (14)	0.0001 (14)
C10	0.0410 (15)	0.0613 (17)	0.0540 (16)	−0.0058 (13)	0.0026 (12)	0.0039 (13)
C11	0.0427 (14)	0.0413 (13)	0.0373 (13)	−0.0026 (11)	0.0090 (11)	0.0029 (10)
C12	0.0480 (14)	0.0402 (13)	0.0314 (12)	−0.0016 (11)	0.0077 (10)	−0.0009 (10)
C13	0.0490 (14)	0.0412 (13)	0.0361 (13)	−0.0059 (11)	0.0073 (11)	−0.0052 (10)
C14	0.0497 (14)	0.0435 (14)	0.0376 (13)	−0.0030 (11)	0.0117 (11)	−0.0006 (11)
C15	0.0682 (18)	0.0477 (15)	0.0384 (14)	−0.0065 (13)	0.0164 (13)	−0.0084 (11)
C16	0.079 (2)	0.0590 (18)	0.0512 (16)	−0.0282 (15)	0.0212 (14)	−0.0149 (13)
C17	0.0654 (18)	0.0560 (16)	0.0424 (15)	−0.0190 (13)	0.0198 (13)	−0.0089 (12)
C18	0.0575 (18)	0.079 (2)	0.0671 (19)	−0.0173 (16)	0.0167 (15)	−0.0032 (16)

### Geometric parameters (Å, °)

**Table:**

N—C1	1.323 (3)	C6—H6	0.9300
N—C4	1.444 (3)	C7—C8	1.372 (3)
N—H1	0.8600	C7—H7	0.9300
O1—C1	1.245 (2)	C8—C9	1.369 (3)
O2—C8	1.375 (3)	C9—C10	1.371 (3)
O2—H2	0.8200	C9—H9	0.9300
O3—C14	1.367 (3)	C10—H10	0.9300
O3—C18	1.417 (3)	C11—C12	1.458 (3)
O4—C15	1.364 (3)	C11—H11	0.9300
O4—H4	0.8200	C12—C17	1.384 (3)
C1—C2	1.479 (3)	C12—C13	1.400 (3)
C2—C11	1.338 (3)	C13—C14	1.371 (3)
C2—C3	1.504 (3)	C13—H13	0.9300
C3—C5	1.521 (3)	C14—C15	1.389 (3)
C3—C4	1.542 (3)	C15—C16	1.369 (3)
C3—H3	0.9800	C16—C17	1.379 (3)
C4—H4A	0.9700	C16—H16	0.9300
C4—H4B	0.9700	C17—H17	0.9300
C5—C6	1.375 (3)	C18—H18A	0.9600
C5—C10	1.389 (3)	C18—H18B	0.9600
C6—C7	1.392 (3)	C18—H18C	0.9600
			
C1—N—C4	114.5 (2)	C7—C8—O2	122.5 (2)
C1—N—H1	122.8	C8—C9—C10	120.4 (2)
C4—N—H1	122.8	C8—C9—H9	119.8
C8—O2—H2	109.5	C10—C9—H9	119.8
C14—O3—C18	117.7 (2)	C9—C10—C5	121.3 (2)
C15—O4—H4	109.5	C9—C10—H10	119.4
O1—C1—N	124.4 (2)	C5—C10—H10	119.4
O1—C1—C2	127.4 (2)	C2—C11—C12	130.9 (2)
N—C1—C2	108.20 (19)	C2—C11—H11	114.6
C11—C2—C1	121.2 (2)	C12—C11—H11	114.6
C11—C2—C3	131.1 (2)	C17—C12—C13	117.9 (2)
C1—C2—C3	107.6 (2)	C17—C12—C11	118.1 (2)
C2—C3—C5	115.6 (2)	C13—C12—C11	124.0 (2)
C2—C3—C4	103.3 (2)	C14—C13—C12	121.0 (2)
C5—C3—C4	111.6 (2)	C14—C13—H13	119.5
C2—C3—H3	108.7	C12—C13—H13	119.5
C5—C3—H3	108.7	O3—C14—C13	125.6 (2)
C4—C3—H3	108.7	O3—C14—C15	114.4 (2)
N—C4—C3	104.2 (2)	C13—C14—C15	120.0 (2)
N—C4—H4A	110.9	O4—C15—C16	118.4 (2)
C3—C4—H4A	110.9	O4—C15—C14	122.0 (2)
N—C4—H4B	110.9	C16—C15—C14	119.5 (2)
C3—C4—H4B	110.9	C15—C16—C17	120.6 (2)
H4A—C4—H4B	108.9	C15—C16—H16	119.7
C6—C5—C10	117.5 (2)	C17—C16—H16	119.7
C6—C5—C3	124.0 (2)	C16—C17—C12	120.9 (2)
C10—C5—C3	118.4 (2)	C16—C17—H17	119.5
C5—C6—C7	121.5 (2)	C12—C17—H17	119.5
C5—C6—H6	119.2	O3—C18—H18A	109.5
C7—C6—H6	119.2	O3—C18—H18B	109.5
C8—C7—C6	119.4 (2)	H18A—C18—H18B	109.5
C8—C7—H7	120.3	O3—C18—H18C	109.5
C6—C7—H7	120.3	H18A—C18—H18C	109.5
C9—C8—C7	119.8 (2)	H18B—C18—H18C	109.5
C9—C8—O2	117.7 (2)		
			
C4—N—C1—O1	174.6 (2)	O2—C8—C9—C10	179.8 (2)
C4—N—C1—C2	−5.3 (3)	C8—C9—C10—C5	0.0 (4)
O1—C1—C2—C11	−7.4 (4)	C6—C5—C10—C9	1.0 (3)
N—C1—C2—C11	172.5 (2)	C3—C5—C10—C9	−178.1 (2)
O1—C1—C2—C3	175.2 (2)	C1—C2—C11—C12	−175.0 (2)
N—C1—C2—C3	−4.9 (2)	C3—C2—C11—C12	1.8 (4)
C11—C2—C3—C5	72.8 (3)	C2—C11—C12—C17	170.9 (2)
C1—C2—C3—C5	−110.1 (2)	C2—C11—C12—C13	−8.2 (4)
C11—C2—C3—C4	−165.1 (2)	C17—C12—C13—C14	−0.9 (4)
C1—C2—C3—C4	12.0 (2)	C11—C12—C13—C14	178.3 (2)
C1—N—C4—C3	12.9 (3)	C18—O3—C14—C13	−0.1 (4)
C2—C3—C4—N	−14.5 (2)	C18—O3—C14—C15	179.8 (2)
C5—C3—C4—N	110.3 (2)	C12—C13—C14—O3	179.0 (2)
C2—C3—C5—C6	7.3 (3)	C12—C13—C14—C15	−0.8 (4)
C4—C3—C5—C6	−110.3 (2)	O3—C14—C15—O4	0.8 (4)
C2—C3—C5—C10	−173.59 (19)	C13—C14—C15—O4	−179.3 (2)
C4—C3—C5—C10	68.8 (2)	O3—C14—C15—C16	−177.9 (2)
C10—C5—C6—C7	−0.9 (3)	C13—C14—C15—C16	2.0 (4)
C3—C5—C6—C7	178.2 (2)	O4—C15—C16—C17	179.8 (2)
C5—C6—C7—C8	−0.3 (3)	C14—C15—C16—C17	−1.5 (4)
C6—C7—C8—C9	1.4 (4)	C15—C16—C17—C12	−0.3 (4)
C6—C7—C8—O2	−179.7 (2)	C13—C12—C17—C16	1.4 (4)
C7—C8—C9—C10	−1.2 (4)	C11—C12—C17—C16	−177.8 (2)

### Hydrogen-bond geometry (Å, °)

**Table:**

*D*—H···*A*	*D*—H	H···*A*	*D*···*A*	*D*—H···*A*
N—H1···O1^i^	0.86	2.09	2.948 (2)	172
O2—H2···O1^ii^	0.82	1.95	2.675 (2)	147
O4—H4···O2^iii^	0.82	2.00	2.721 (2)	147

Symmetry codes: (i) −*x*, −*y*+1, −*z*+1; (ii) −*x*, *y*+1/2, −*z*+1/2; (iii) −*x*+1, −*y*+1, −*z*.
